# Human Activity Recognition Method Based on FMCW Radar Sensor with Multi-Domain Feature Attention Fusion Network

**DOI:** 10.3390/s23115100

**Published:** 2023-05-26

**Authors:** Lin Cao, Song Liang, Zongmin Zhao, Dongfeng Wang, Chong Fu, Kangning Du

**Affiliations:** 1 The Key Laboratory of Information and Communication Systems, Ministry of Information Industry, Beijing Information Science and Technology University, Beijing 100101, China; charlin@bistu.edu.cn (L.C.); liangsong1998@163.com (S.L.); kangningdu@bistu.edu.cn (K.D.); 2 The Key Laboratory of the Ministry of Education for Optoelectronic Measurement Technology and Instrument, Beijing Information Science and Technology University, Beijing 100101, China; 3 Beijing TransMicrowave Technology Company, Beijing 100080, China; wdf@tsmtc.com; 4 School of Computer Science and Engineering, Northeastern University, Shenyang 110169, China; fuchong@mail.neu.edu.cn

**Keywords:** human activity recognition, attention mechanism, multi-domain feature fusion, multi-classification focus loss, FMCW radar sensor

## Abstract

This paper proposes a human activity recognition (HAR) method for frequency-modulated continuous wave (FMCW) radar sensors. The method utilizes a multi-domain feature attention fusion network (MFAFN) model that addresses the limitation of relying on a single range or velocity feature to describe human activity. Specifically, the network fuses time-Doppler (TD) and time-range (TR) maps of human activities, resulting in a more comprehensive representation of the activities being performed. In the feature fusion phase, the multi-feature attention fusion module (MAFM) combines features of different depth levels by introducing a channel attention mechanism. Additionally, a multi-classification focus loss (MFL) function is applied to classify confusable samples. The experimental results demonstrate that the proposed method achieves 97.58% recognition accuracy on the dataset provided by the University of Glasgow, UK. Compared to existing HAR methods for the same dataset, the proposed method showed an improvement of about 0.9–5.5%, especially in the classification of confusable activities, showing an improvement of up to 18.33%.

## 1. Introduction

HAR is a significant research area in artificial intelligence, with broad applications in human–computer interaction, intelligent surveillance, and other fields. HAR primarily acquires information about human targets through cameras or sensor devices and employs machine learning [1] or deep learning algorithms [2]. Currently, wearable electronic devices, cameras, radars, and other devices are the mainstream devices used for HAR [3,4,5].

Wearable electronic device sensors have the potential to acquire a vast amount of information about human movement [6]. However, these sensors have limitations as they must be attached to the human body, and individuals must wear them at all times for proper use. In contrast, image-based HAR primarily relies on cameras or other image-acquisition devices to obtain information about human activities [7]. High-resolution cameras can accurately identify human activities, but they have limitations in functioning under all-round and all-weather conditions, regardless of the environmental conditions. Additionally, the use of image-based techniques can compromise privacy.

In comparison, FMCW radar sensors can overcome and improve upon the limitations of the devices mentioned above. An FMCW radar sensor can guarantee users’ privacy and has the advantage of working under any lighting conditions, as well as having the ability to function in all-weather, even in various harsh environmental conditions such as fog and smoke [8,9]. Therefore, FMCW radar sensors are widely used in the application fields of HAR, including urban military activity monitoring, elderly care, and automatic driving [10,11,12].

In urban security systems, HAR using an FMCW radar sensor is a crucial task. FMCW radar sensors can be used for long-distance pedestrian recognition [13] and HAR for short distances [12], including identifying dangerous activities such as boxing and jumping with guns [10]. In indoor elderly safety care, FMCW radar sensors can accurately recognize human activities while preserving privacy. For instance, Abdu et al. proposed a fall detection system that accurately determines whether a person has fallen based on radar image classification. This system was specifically designed to assist older people [14].

Typically, FMCW radar sensors can extract human point cloud data [15] or 2-D domain spectra [16] to determine the target’s activities. However, using the multi-frame point cloud accumulation method has limitations because some activities may have very similar point cloud results, such as sitting down and standing up, which are opposite activities. In contrast, extracting 2-D domain spectra from FMCW radar sensor data allows for more comprehensive data collection, and these feature maps can be viewed as 2-D images [16,17] or time series [18,19]. Various deep-learning methods can use these maps to classify and recognize human activities. 

Because human activities involve multiple dimensions and require considering several target feature information simultaneously, a single 2-D domain spectrum cannot fully characterize human activities. Consequently, many researchers have turned to feature fusion networks, which fuse at least two 2-D domain spectra to achieve HAR. These methods typically include multi-sensor fusion [20,21,22,23,24] and multi-domain feature fusion [25,26,27,28,29,30]. Li et al. used one FMCW radar and three ultra-wideband pulsed radar sensors to collect human gait information simultaneously and employed signal-level fusion and decision-level fusion methods [20]. Gorji et al. installed two FMCW radar sensors on indoor ceilings and walls to collect human activity data [21]. In their research, refs. [22,23,24] used wearable and radar sensors to acquire human activity data. They employed support vector machines (SVM) or bidirectional long short-term memory (Bi-LSTM) to complete feature fusion methods that combined data from both types of sensors. These techniques led to an improvement in the accuracy of HAR. Although multi-sensor fusion methods have effectively enhanced HAR accuracy, they are generally more complex and expensive to process.

Using a single radar sensor, the multi-domain feature fusion network (MFFN) can also improve HAR accuracy. Arab et al. proposed a two-channel CNN (Convolutional Neural Network) for HAR that utilizes a 1-D CNN and a 2-D CNN for feature extraction and fusion of the original signal and TD maps, respectively [25]. The literature proposed different feature maps for different activity types to achieve HAR [26,27,28,29]. Zhang et al. used splicing of spectrograms amplitude and phase of human activity as the input features of the network [26]. Li et al. fused different combinations of TD maps, cadence velocity diagrams (CVD), and TR maps of human activities [27]. The MFFNs for human activity with TR, TD, and range-Doppler (RD) maps were used to accomplish HAR [28,29]. The most commonly used data in the MFFN are TR and TD maps. These two kinds of data have strong complementarity since they simultaneously consider information such as the position and velocity of the target in 3D space. Therefore, we have chosen these two kinds of features as the input of the MFFN. 

However, the TD and TR domain feature maps have different strengths and weaknesses in discriminating between human activities. For instance, TD maps are effective in specific activities involving noticeable velocity changes, such as falling, but may misclassify activities such as drinking or picking up an object. Conversely, the misclassification rate for activities such as sitting down, picking up an object, or drinking is lower in the TR maps. Although the two types of data can complement each other’s information through the MFFN, it is crucial to design a neural network architecture that is more suitable. Therefore, this paper proposes a network that combines two types of features from different depth levels to enhance the accuracy of HAR. In addition, the commonly used abbreviations in this article are listed in Table A1 in Appendix A for reference.

The main contributions of this paper are:

- We propose the multi-domain feature attention fusion network (MFAFN) model for HAR based on the FMCW radar sensor, which enhances the VGG13 architecture by fusing TR and TD maps as the multi-domain feature fusion baseline network (MFFBN) model. Specifically, we introduce the MAFM to more comprehensively unite the 2-D domain spectrum by combining single-domain shallow, medium, and deep attention-weighted features;
- We replace the traditional cross-entropy loss function with a multi-classification focus loss (MFL) function to improve the weight of confusable samples in the MFFBN and the MFAFN models;
- We evaluate the effectiveness of our proposed algorithm on a publicly available dataset and found that it slightly improves the recognition accuracy compared to existing methods for HAR.

## 2. Related Work

This section provides a comprehensive review of prior work on HAR using radar sensors, divided into three parts: HAR based on single-domain features, HAR based on multi-domain feature fusion methods, and HAR based on attention mechanisms.

### 2.1. HAR Based on Single-Domain Features

In the field of HAR, researchers commonly utilize TD maps as the 2-D domain spectrum to classify. Taylor et al. combined three machine learning and three deep learning algorithms with principal component analysis (PCA) to implement HAR using TD maps as input. The best results were achieved by combining CNN and PCA in the results of six methods [11]. Saeed et al. used a ResNet network to classify TD maps for six activities: falling, sitting down, standing up, walking, drinking, and picking up an object. They achieved 100% accuracy for falling activity recognition by using TD maps for classification [17]. Zhu et al. proposed a deep learning model that combines 1D-CNN and long short-term memory (LSTM) [18]. Shrestha et al. presented a recurrent network architecture based on LSTM and Bi-LSTM [19]. Both studies treat TD maps as time series rather than 2-D images, which differs from how CNNs operate. However, it should be noted that training an LSTM model takes longer [18,19].

### 2.2. HAR Based on Multi-Domain Feature Fusion Method

When implementing HAR using radar sensors, the MFFNs can be divided into feature-level fusion networks [28,29,30,31,32,33,34,35] and decision-level fusion networks [36,37,38]. Feature-level fusion methods extract features from multiple inputs and combine them to create an even more comprehensive and richer feature representation. For example, simple stitching operations on features [29,30,31,32,33,34,35] and feature fusion summation operations [28] are the most common methods.

Wang et al. utilized a graph convolutional network (GCN) to fuse TR, TD, and RD maps of human activities and then performed HAR in graph classification [29]. Bai et al. proposed a dual-channel deep convolutional neural network (DCNN) based approach for radar-based human gait recognition. They fused two TD maps generated using short-time Fourier transform (STFT) with different sliding window sizes to achieve fine human gait recognition [30]. Jia et al. extracted hand-crafted feature maps, phase maps, and TD maps of human activities and fused them using SVM, resulting in two and three map fusions, respectively. The experimental results revealed that the fusion of hand-crafted features and TD maps led to better performance [31].

Numerous MFFNs have been proposed for HAR based on various radar 2-D domain spectral features. Zhao et al. extracted TD maps and CVD of human activities. They fused both feature maps using the CentralNet network, which links the relationship between the two features and more effectively combines TD maps and CVD. However, this approach is only suitable for feature fusion with correlated features [32]. In another study, Chen et al. designed a pre-trained MobileNetV3 lightweight network model and a feature pyramid network (FPN) based multiscale feature extraction model to overcome the challenge of insufficient data [33]. A hierarchical fusion network (HFN) with multi-domain features was proposed in the literature using narrowband radar. The HFN contains an intra-domain network and an inter-domain network. The intra-domain network reduces redundant features, and the inter-domain network fuses high-level features from different domains [34]. Helen et al. proposed a tower CNN that inputs three channels of red-green-blue (RGB) TD maps of human activities separately, with each color channel image as a parallel input layer. They utilized a splicing operation after the convolutional module and then learned the fused features using a 7-layer dense neural network [35]. Ding et al. proposed a novel MFFN model with a summation approach. To extract features, a combination of a 1-D CNN and LSTM network is utilized for TR and TD maps, while a 2-D CNN is employed for RD maps. Finally, the three 2-D domain spectra are fused using adaptive weight fusion, and the network model effectively classifies activities [28].

In addition, decision-level fusion is also utilized by training separate models for each 2-D domain spectrum and then combining the results via voting. Chen et al. conducted six preprocessing steps for the TD maps of human activities and utilized CNN models with different training architecture parameters for each feature. They implemented a weighted voting method to fuse the information, and the weight matrix was estimated based on the classification results of the training dataset. This method ultimately improved HAR accuracy effectively [36]. Jokanovic et al. used a stacked autoencoder-based feature extraction for each feature, including the TD, TR, and RD domains. The three parts were combined via weighted voting to obtain activity classification results [37]. Kim et al. proposed a range-distributed convolutional neural network (RD-CNN) architecture for HAR by combining range-time-Doppler (RTD) maps [38]. The TD map of the range dimension is utilized as input to the network. The final classification result is obtained by calculating the sum of probabilities across the range dimensions. In summary, there are various MFFN methods for HAR based on radar sensors, including feature-level fusion and decision-level fusion. These methods combine multiple 2-D domain spectral features to achieve better performance in HAR. However, traditional neural networks treat all inputs equally, which may not effectively distinguish different input information. The introduction of an attention mechanism can help neural networks focus more precisely on the parts that have a greater impact on the results, thus improving the model’s performance.

### 2.3. HAR Based on Attention Mechanism

Recent literature has explored the use of attention mechanisms to enhance the accuracy of HAR models. Abdu et al. employed AlexNet and VGGNet networks to extract TD maps of human activities, respectively [14]. They proposed an efficient channel attention module to enhance the extraction process’s efficiency. Finally, the canonical correlation analysis (CCA) module was used for feature fusion. DU et al. utilized a feature refinement module based on channel and spatial attention to enhance the accuracy of multi-channel feature fusion for HAR [39]. A method was proposed in [40] to extract TD maps of human activities. It combines a multi-head attention mechanism, which captures global information from TD maps, with locally extracted feature maps from a convolutional auto-encoder (CAE). The introduction of the multi-head attention mechanism resulted in higher classification accuracy.

The literature mentioned above faces two main issues. Firstly, the attention mechanism approach only operates on a single layer of features and does not account for the relationship between multiple layers of features. Secondly, accurate recognition of human activity based on FMCW radar sensors is highly challenging, mainly due to the high similarity between confusable activities. Although using multiple sensors or radar 2-D domain spectra can effectively improve the accuracy of HAR by exploiting complementary information, which needs to address the issue of confusable activities. Therefore, in this study, we propose the MFAFN model that addresses the abovementioned issues. The network first incorporates attention-weighted features from single-domain features at shallow, middle, and deep layers. Then, the fusion is completed for the two-domain features after a layer of pooling. Instead of the conventional cross-entropy loss function, the MFL function is utilized. This approach reduces the weights of easily classified samples while increasing the importance of easily confusable samples, resulting in improved accuracy in HAR.

## 3. HAR System Overview

**Figure 1:**
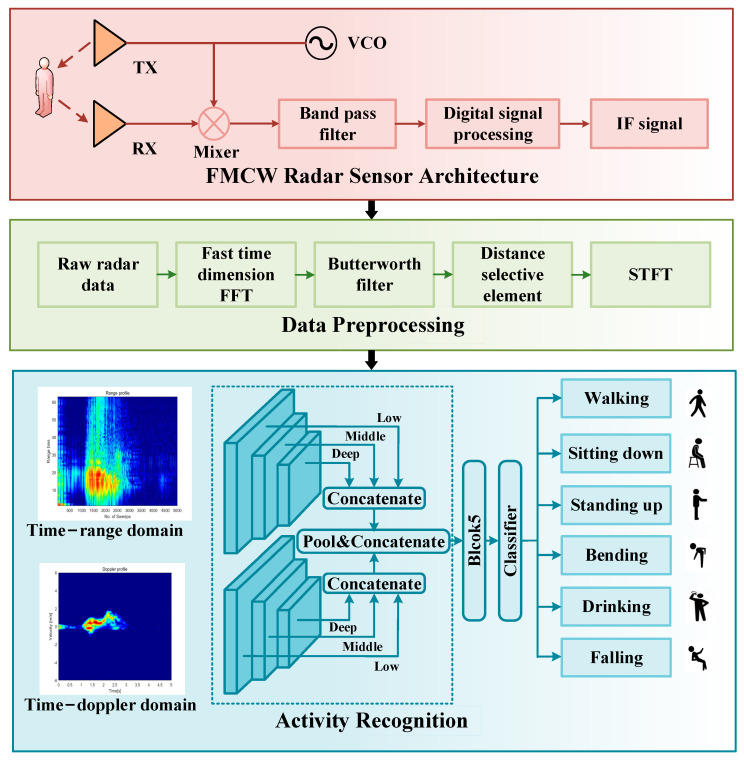
HAR System Overview.

This section presents an overview of the proposed HAR system using the MFAFN model, as shown in Figure 1. The system consists of three main parts: the FMCW radar sensor architecture, the data preprocessing phase, and the activity recognition phase. The FMCW radar sensor architecture and the data preprocessing phase can be collectively referred to as the 2-D domain spectra extraction process. The activity recognition phase in this section focuses on the convolutional neural network and the attention mechanisms used. The dashed part in Figure 1 indicates the innovative part of the proposed method in this paper, which will be described in detail in Section 4.

### 3.1. 2-D Domain Spectra Extraction

This section describes FMCW radar sensor architecture and data preprocessing, as illustrated in Figure 1. The FMCW radar sensor transmits a continuous frequency-modulated signal to the target through the TX antenna and receives the reflected signal from the target through the RX antenna. The intermediate frequency (IF) signal is then converted into a digital signal by an analog-to-digital converter (ADC) in the radar equipment, which undergoes several digital signal processing (DSP) steps to obtain the raw radar data.

To obtain a 2-D spectrogram that can more accurately represent information on human activity, further processing of the raw radar data is necessary. Firstly, a TR map is obtained by performing a fast Fourier transform (FFT) on the raw radar data, which is a matrix of size sampling point and chirp. A Butterworth high-pass filter is applied to this TR map to remove noise. As human activity is a signal collected within a specific range from the radar, the range selection unit needs to be manually set to select the primary range of human activity. Subsequently, the TD map characterizing people’s activity is extracted using the short-time Fourier transform (STFT) [41]. The STFT determines the analysis period by selecting the window function, and the spectrum at different time intervals is obtained by moving the window function graph. Ultimately, the spectrogram is expressed as follows:

(1) $$S(m,n)={\left|X(m,n)\right|}^{2}={\left|\sum_{k=0}^{N-1}x(k)\omega (k-m)e^{\left(-j2\pi kf\right)}\right|}^{2},$$

where m represents the time index, n represents the frequency index, ω(·) denotes the window function, and N denotes the length of the window.

### 3.2. Convolutional Neural Network

CNNs have shown excellent results in various computer vision tasks. Since the emergence of the AlexNet network in 2012 [42], many CNNs have been developed, including VGGNet [43], GoogleNet [44], and others. These models have demonstrated impressive results in image classification.

In the field of HAR based on FMCW radar sensors, the 2-D domain spectrum is commonly utilized as a feature for activity classification. This feature map can be viewed as images with different-sized pixel values. As a result, CNNs are suitable for implementing HAR using radar 2-D domain spectrum.

We have chosen the VGGNet network model as the reference model for our research. The VGGNet model was the runner-up of the ImageNet competition in 2014 and had lower model complexity than GoogleNet, which has the highest classification accuracy. Because the HAR dataset based on the FMCW radar sensor is less affluent than the image domain, it is unsuitable for complex network models with smaller datasets. To design the baseline model for the MFAFN model, we chose the VGG13 model, which is one of the models in the VGGNet group.

### 3.3. Attention Mechanism

Typically, deeper neural network structures often lead to better performance, but at the cost of increased time and memory consumption. We have tackled these issues by utilizing an attention mechanism that concentrates on the most informative regions of the input data. Specifically, we utilize the SENet architecture [45], which has low computational complexity and is widely used in practice. The output of the attention mechanism is a probability map that assigns different weights to different regions of the input features, depending on their importance for the task of HAR.

**Figure 2:**
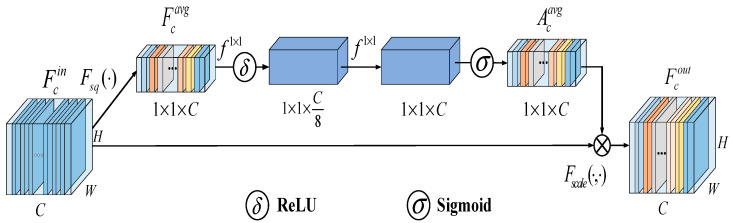
SENet attention mechanism module.

Figure 2 illustrates that the input feature assumes a feature channel of C, a width of W, and a height of H, denoted as $F_{c}^{in}$. Initially, the feature channels undergo compression using the squeeze operation. The next step involves converting the 2-D features of each channel into actual numerical values. This process generates a feature map $F_{c}{}^{avg}$ with a size of 1 × 1 × C.

(2) $$F_{c}{}^{avg}=F_{sq}(F_{c}^{in})=\frac{1}{W\times H}\sum_{i=1}^{W}\sum_{j=1}^{H}F_{c}^{in}(i,j),$$

where $F_{c}{}^{avg}$ denotes the result of applying global average pooling to the input features. After the squeeze operation, we use the excitation operation to generate weights for each feature channel. Instead of a fully connected layer, we employ a 1 × 1 convolutional layer to reduce the parameters and computational costs. The channel compression rate is set to eight to achieve this reduction. The weight $A_{c}^{avg}$ for each feature channel is calculated as follows:

(3) $$A_{c}^{avg}=\sigma (f^{1\times 1}(\delta (f^{1\times 1}(F_{c}^{avg})))),$$

where $A_{c}^{avg}$ denotes attention weight, δ(·) denotes rectified linear unit (ReLU) activation function. σ(·) represents the sigmoid function, and $f^{1\times 1}$ represents the 1 × 1 convolution operation. Finally, the weighting operation is performed, where the weights generated in the previous step are weighted channel by channel into the input features $F_{c}^{in}$ to obtain $F_{c}^{out}$:

(4) $$F_{c}^{out}=F_{scale}(A_{c}^{avg},F_{c}^{in})=A_{c}^{avg}\cdot F_{c}^{in},$$

where $F_{c}^{out}$ denotes the attention-weighted features.

## 4. HAR Architecture Based on the Multi-Domain Feature Attention Fusion Network

**Figure 3:**
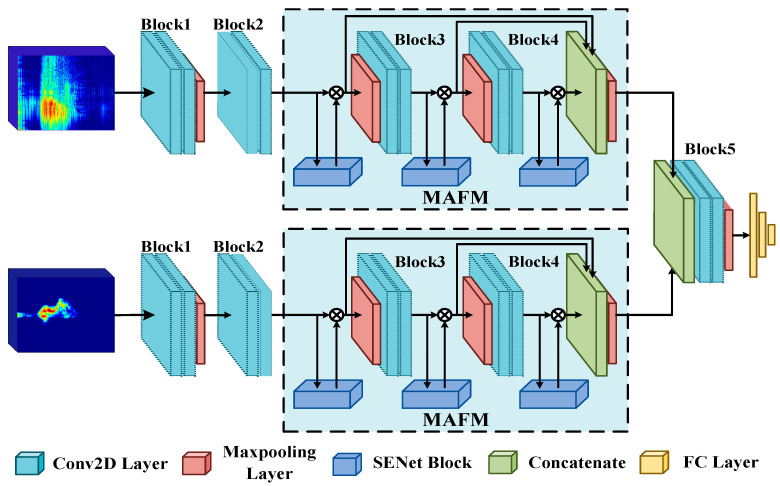
The MFAFN model architecture.

This section details the MFAFN model, whose network architecture is illustrated in Figure 3. The model requires two inputs, TR and TD maps. They are treated as optical images with distinct pixel values, serving as inputs to our proposed HAR model. The TR map effectively captures changes in range over time as the human body moves, making it suitable for distinguishing activities characterized by significant alterations in range. In contrast, in situ activities tend to exhibit minimal range changes, which can lead to confusion between different activities. In contrast, the TD map depicts variations in velocity for each activity throughout the temporal sequence, making it proficient in distinguishing activities that involve pronounced speed changes. However, activities with similar speed alterations may also cause confusion when relying solely on the TD map.

To comprehensively characterize human activities, we incorporate both the range and velocity information of the target. By simultaneously analyzing and learning from the TR and TD maps, our HAR model extracts motion features and accomplishes HAR. We emphasize that the TR and TD maps play crucial roles in the HAR model, effectively fusing the two features and resulting in higher accuracy in HAR.

Its backbone network follows a symmetric structure designed based on the VGG13 network. In addition, the model incorporates the SENet attention mechanism module, which is applied after the second convolutional layer following the three blocks. The process of feature fusion involves combining three attention-weighted features and using a pooling layer to preserve salient feature information. The fused features are then further extracted using two convolutional layers and an additional pooling layer. Finally, the model’s feature vector output is fed into a classification module consisting of fully connected and softmax layers, which classifies the features and produces the HAR results. This section introduces three components of the MFAFN model: the multi-domain feature fusion baseline network, the multi-feature attention fusion module, and the multi-classification focus loss function.

**Figure 4:**
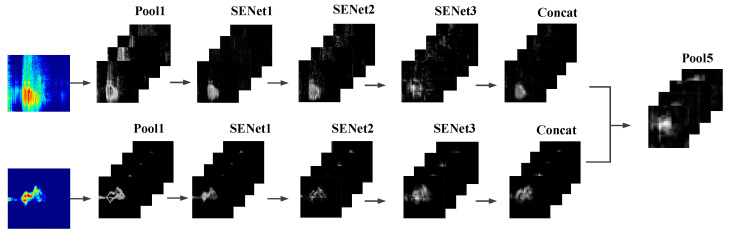
Visualization of feature maps.

As shown in Figure 4, we visualize the features at the following stages: after the first pooling layer, after the first, second, and third attention mechanisms, after the first concatenation operation, and after the fifth pooling layer. For each visualization, we arrange the data from four channels.

### 4.1. Multi-Domain Feature Fusion Baseline Network

**Figure 5:**
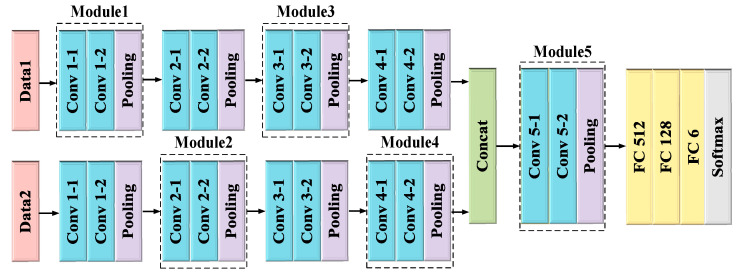
The MFFBN model architecture.

Figure 5 illustrates the proposed MFFBN model, which consists of two input channels for TR and TD maps. The network has a symmetric structure, and we present only the details of the first channel network. The network comprises five modules, each consisting of two convolutional layers and one pooling layer. The convolutional kernel size in the network is 3 × 3 with a step size of 1. Each convolutional operation reduces the channel size by half. The pooling layer window size is set to 2 × 2 with a step size of 2, reducing the image size by half after each pooling operation. These five modules differ only in the number of convolutional kernels, which are 32, 64, 128, 256 and 512, respectively.

To prevent gradient explosion and disappearance, a Batch Normalization (BN) layer is included after each convolutional layer. This also helps to speed up the training and convergence of the network. Additionally, the ReLU activation function is applied to all convolutional layers:

(5) f(z)=max(0,z),

where f(z) equals to input z when z is greater than 0, and 0 otherwise. The input image is in RGB format with 224 × 224 pixels, and the first module generates a feature map with 32 channels and a size of 112 × 112 pixels. Each subsequent module doubles the number of channels compared to the previous module and reduces the image size by half. After the fourth module, a feature map with 256 channels and a height of 14 × 14 pixels is produced. A cascade operation is then applied to channels one and two, as represented by:

(6) $$F_{fuse}=Con(F_{TR}(x),F_{TD}(x)),$$

where Con(·) represents the splicing operation, $F_{TR}(x)$ denotes the TR map of the fourth output module, and $F_{TD}(x)$ represents the TD map of the fourth output module. After the fifth module, a feature map with 512 channels and a size of 7 × 7 pixels is outputted for the fused features. The fully connected layers’ size is 512, 128, and 6. To avoid overfitting caused by the deep network, we add a Dropout layer with a parameter of 0.5 to each fully connected layer. Dropout randomly deletes some hidden neurons to improve the model’s generalization ability effectively.

The final layer of the network is the softmax layer, which aims to maximize prediction accuracy by calculating the loss between the predicted data and the actual label. The class probabilities are then calculated as follows:

(7) $$a_{k}=P(C|x,W)=\frac{e^{z_{k}}}{\sum_{i=1}^{K}e^{z_{i}}},k=0,1,\ldots K-1,$$

where $a_{k}$ represents the predicted probability of each classification, C represents the set of categories, W denotes the weight vector, and $z_{k}$ denotes the value obtained by linearly weighting the features of the sample x.

### 4.2. Multi-Feature Attention Fusion Module

To make the model adaptively focus on significant target signal regions and make better use of features, we incorporate an attention mechanism into the MFFBN. This mechanism enables the model to focus on the essential features of the 2-D domain spectrum, reduce feature redundancy by reassigning feature weights, and use feature information more efficiently. In this study, we utilize the SENet channel attention mechanism to focus on regions essential for human activity. The SENet employs a Squeeze-and-Excitation (SE) module architecture to dynamically determine the relevance of each feature map. This is achieved by computing the attention weights for each channel and using these weights to scale the input features.

Low-level features generally have high resolution and more detailed information but weak semantic information. In contrast, high-level features contain more semantic information but have lower resolution and less detailed information. Although the semantic information of the bottom-level features is weaker, it is still significant. In contrast, the information in higher-level features may lose the most detailed information due to the deeper convolution layers and smaller feature dimensions. We propose the MAFM to enhance the feature representation of HAR. The MAFM combines low-level, mid-level, and high-level features and applies a channel attention mechanism to each depth level of features, allowing the model to focus on the essential features before fusing them at different levels. This approach enables the model to more effectively utilize the different types of features at varying levels of complexity.

The SENet module is presented in Figure 2 and implemented using a series of operations, including a global average pooling layer, two convolutional layers with a kernel size of 1 × 1, a ReLU activation function, and a sigmoid function. As shown in Figure 3, the attention maps are obtained after the second convolution of the second, third, and fourth blocks of the MFFBN model, respectively. We then adjust the three attention-weighted features to 64 × 64 pixels and connect them. The optimized feature maps can be expressed as follows:

(8) $$F^{\prime }(x)=(Con(F_{L}(x)\cdot A_{L}(x),F_{M}(x)\cdot A_{M}(x),F_{H}(x)\cdot A_{H}(x))),$$

where $F_{L}(x)$, $F_{M}(x)$, and $F_{H}(x)$ represent the low-level, mid-level, and deep-level features, respectively, and $A_{L}(x)$, $A_{M}(x)$, and $A_{H}(x)$ denote the corresponding attention maps.

After fusing the TR and TD maps with a layer of pooling, we can express this module as:

(9) $$F_{fuse}^{\prime }=Con(pool(F_{TR}^{\prime }),pool(F_{TD}^{\prime })),$$

where pool denotes the maximum pooling layer, $F_{TR}^{\prime }$ and $F_{TD}^{\prime }$ denote the multi-attentive weighted features of TR and TD maps, respectively, and $F_{fuse}^{\prime }$ denotes the fused features.

### 4.3. Multi-Classification Focal Loss Function

Based on FMCW radar sensors, HAR can be challenging in distinguishing between some activities due to slight differences in their 2-D domain spectra. To address this issue, we apply the multi-classification focal loss function [46]. The focal loss function was initially developed for target detection to solve the classification imbalance problem. The traditional cross-entropy loss function treats all samples equally, leading to high error rates in identifying complex classification samples. The focal loss function introduces a moderator that reduces the weight of easy-to-classify samples and emphasizes the importance of confusable samples. Therefore, the focal loss function is suitable for classifying confusable samples.

The focal loss function dynamically adjusts the weights of the loss function based on the difference between the predicted probability and actual label of each sample, rather than using fixed weights. If a sample is correctly classified, its weight decreases. Conversely, if it is misclassified, its weight increases. Specifically, by introducing the modulation factor denoted by ${\left(1-p_{i}\right)}^{\gamma }$, the MFL function can be expressed as:

(10) $$L_{mfl}=-{\sum_{i=1}^{N}\left(1-p_{i}\right)}^{\gamma }t_{i}\log(p_{i}),$$

where, N represents the number of categories, $p_{i}$ is the predicted probability, and $t_{i}$ = 1 if i belongs to the actual label, otherwise, $t_{i}$ = 0. The focus parameter γ is used to control the rate of reducing the weight of easy-to-classify samples. When γ = 0, the MFL function is equivalent to the multi-classification cross-entropy function. By setting γ equal to 2, we adjust the weight of the loss function to increase the model’s sensitivity to confusable samples. When samples are misclassified and $p_{i}$ is small, the modulation factor almost tends to 1, so the loss is not affected. When $p_{i}$ is close to 1, the modulation factor almost tends to 0, and the loss of easy-to-classify samples is weighted down. 

In the focal loss factor, the most commonly used values are usually 1.5 and 2. Table 1 performed a comparative analysis by using three different values. It can be seen that the performance is best when γ = 2.

In summary, the flow of the MFAFN model algorithm is shown in Algorithm 1.


**Algorithm 1 MFAFN Model**
     **Input:** TR and TD maps;     **Output:** Label: 0–5 (six types of activities);     **for all** training images **do**        1. Input TR and TD maps into the first block of the network and obtain the respective characteristics;        2. Apply the channel attention mechanism after the second layer of convolution for the second, third, and fourth blocks, resulting in attention maps for each block (denoted as $A_{L}(x)$, $A_{L}(x)$, $A_{L}(x)$);        3. Resize the three features of the two domains to 64 × 64 and concatenate them to obtain the multi-attentive weighted features (denoted as $F_{TR}^{\prime }$ and $F_{TD}^{\prime }$), respectively;        4. After a layer of pooling, concatenate the multi-attentive weighted features of the two domains to obtain $F_{fuse}$;        5. Obtain deep features $F_{fuse}$ by passing the input through block5, and classify the input by feeding the features into a fully connected layer followed by a softmax layer;        6. Calculate the MFL based on the predicted and true values, and perform backpropagation to update the network parameters.

## 5. Experimental Results and Analysis

### 5.1. Experimental Setup

#### 5.1.1. Dataset Description

The dataset used in this experiment was provided by the University of Glasgow, UK [47]. It was acquired by a C-band 5.8 GHz FMCW radar sensor and consisted of six human activities, including walking, sitting down, standing up, picking up an object, drinking, and falling.

**Figure 6:**
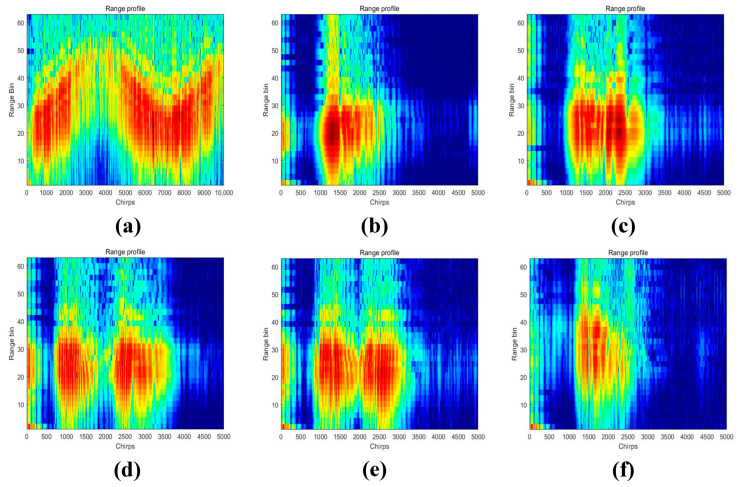
TR maps of six human activities. (**a**) Walking. (**b**) Sitting down. (**c**) Standing up. (**d**) Picking up an object. (**e**) Drinking. (**f**) Falling.

**Figure 7:**
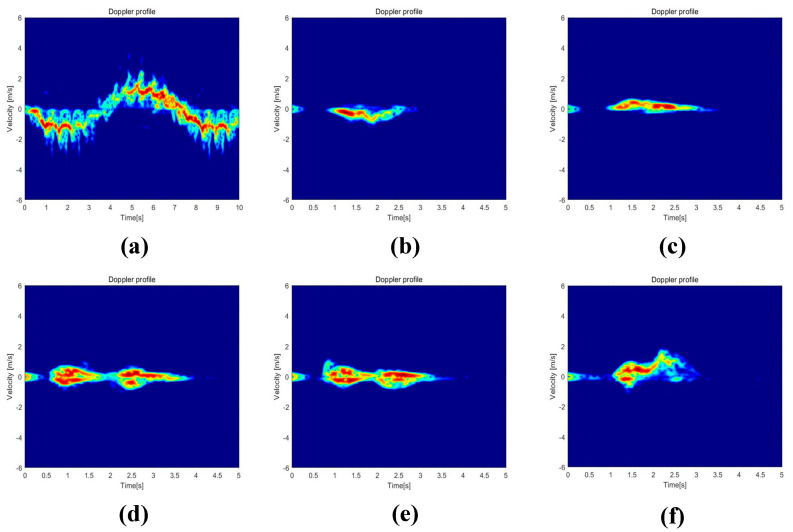
TD maps of six human activities. (**a**) Walking. (**b**) Sitting down. (**c**) Standing up. (**d**) Picking up an object. (**e**) Drinking. (**f**) Falling.

MATLAB tools were utilized to preprocess the raw radar data of this dataset, resulting in the TR and TD maps of the six human activities. The numerical matrices were then converted to RGB image format, and the values of the matrix elements were used as the pixel values of the images. The proposed network model was applied for classification, resulting in a dataset of 1338 images. Figure 6 shows the six human activities’ TR maps, Figure 7 shows the six human activities’ TD maps.

#### 5.1.2. Environment Settings

The experimental setup for radar data preprocessing involved both hardware and software components. For hardware, an NVIDIA GeForceRTX3080 graphics card was used on a Linux system running Ubuntu 18.04LTS. The computer was equipped with an Intel(R) Core (TM) i5-6300HQ CPU for processing. For software, the experiments were conducted on MATLAB version 2020a, which is a commercial mathematical software developed by MathWorks, a company based in the United States. And the data processing was implemented in Python 3.9 using Pytorch 1.13. To improve the computation speed, a CUDA11.0 parallel computing architecture was employed.

During the training process, the Epoch was set to 50 for training, with a batch size of 32. The stochastic gradient descent (SGD) optimization algorithm was used, with a momentum of 0.9 and a weight decay parameter of 0.0005. We used a learning rate optimization strategy to speed up convergence and improve the model’s generalization ability. This strategy allows the model to find the optimal global solution more efficiently while enabling finer adjustment of the model parameters later for better convergence. The initial value of the learning rate was set to 0.0001, and it grew linearly according to the step size until it reached the maximum value of the learning rate of 0.005, after which the slow-heating phase of the learning rate was completed. The learning rate in the decreasing phase utilized an exponential decay strategy.

For the dataset, the data sets of the two domains were randomly divided into five equal subsets based on the activity classification. In each iteration of the 5-fold cross-validation, four subsets were used as the training set, and one subset was used as the test set. All experimental results reported in this paper are based on the values obtained from the 5-fold cross-validation process.

### 5.2. Experimental Results and Analysis

To verify the effectiveness of the proposed method, we conducted experiments comparing the MFFBN model with the single-domain feature network (SFN), and an ablation experiment by adding the MFL function and the MAFM in the MFFBN model. Additionally, the proposed method is compared with other HAR methods.

#### 5.2.1. Assessment Indicators

Five evaluation metrics, including accuracy, Recall, Precision, F1-Score, and confusion matrix, were utilized to evaluate the performance of the proposed model in this study. All experimental results were obtained by testing on the same dataset. The metrics are defined as follows:

(11) $$\mathrm{Accuracy}=\frac{TP+TN}{TP+FN+TN+FP},$$

(12) Recall=TPTP+FN,

(13) $$Precision=\frac{TP}{TP+FP},$$

(14) F1−Score=2(Precision×Recall)Precision+Recall,

where TP and TN represent the number of correctly and incorrectly predicted samples among positive cases. While FP and FN represent the number of correctly and incorrectly predicted samples among negative cases. 

The Recall rate measures the percentage of correctly predicted positive class data in the dataset out of all positive class data. The Precision rate measures the percentage of positively predicted data in the dataset out of all optimistic predicted data. The F1-Score is the weighted average of the Precision and Recall rates.

In addition, the performance metrics of individual activities are shown through the confusion matrix, also known as the error matrix. Displaying the confusion matrix as a visual graph provides a clearer view of the model’s classification results for each activity. The column elements of the confusion matrix represent the actual activity type, and the row elements represent the predicted activity type. Specifically, the walking, sitting down, standing up, picking up an object, drinking, and falling activities correspond to labels A1, A2, A3, A4, A5, and A6, respectively.

#### 5.2.2. Comparison Experiment between the MFFBN and the SFN Models

This section compares the effectiveness of the MFFBN and the SFN models. The SFN model uses the VGG13 network, while the MFFBN model is an improved VGG13 network that includes features from two domains.

Table 2 shows that the recognition accuracy of TD maps for human activities can reach 92.12%, while the recognition accuracy of TR maps can only reach 75.08%. The recognition accuracy of the MFFBN model for human activities is 93.1%. These results show that the TD maps possess a higher confidence level than the TR maps, and the fusion of TR and TD features can compensate for the shortage of single-feature recognition.

**Figure 8:**
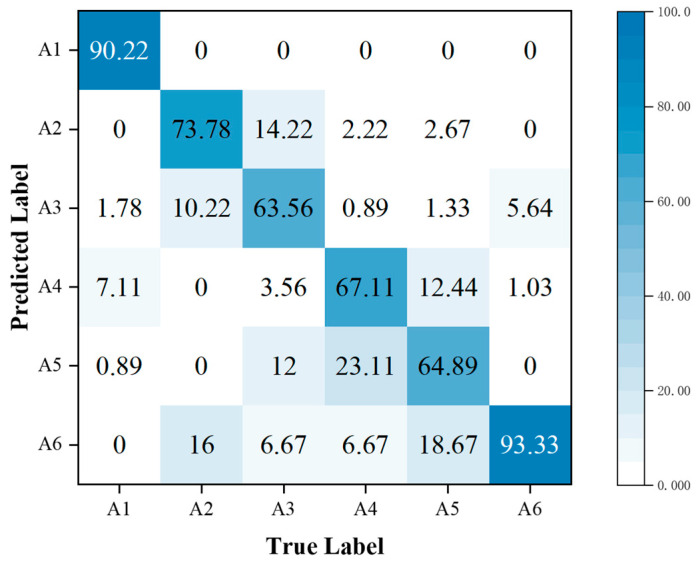
Confusion matrix for classification accuracy of the SFN model using TR maps.

**Figure 9:**
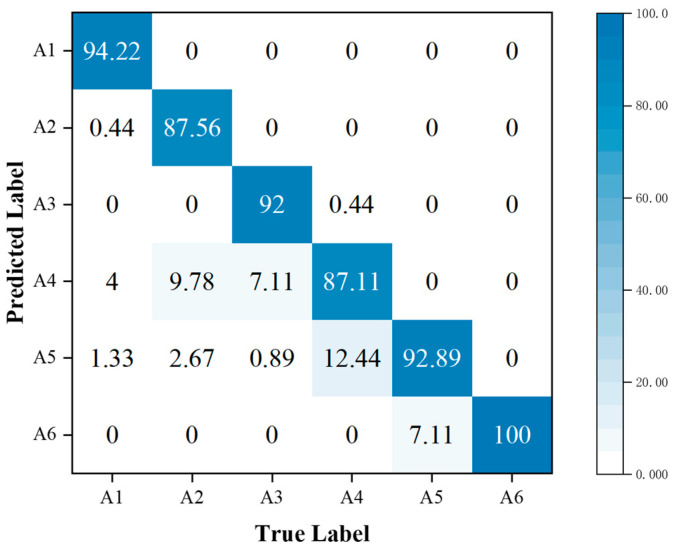
Confusion matrix for classification accuracy of the SFN model using TD maps.

To further analyze the correct and error rates for each activity in the three sets of experiments, confusion matrices were generated for the SFN model using TR maps, the SFN model using TD maps, and the MFFBN model. Figure 8 and Figure 9 demonstrate that the recognition accuracy for standing, picking up an object, and drinking activities is lowest for the TR map. The TD maps have the lowest recognition accuracy for picking up an object and sitting down activities. Overall, the TD maps have higher accuracy in recognizing all activities other than the TR maps. However, the TR maps exhibit lower probabilities of misclassifying sitting down activities as picking up an object and drinking activities, respectively. The two domain feature maps have different advantages for recognizing the six activities.

**Figure 10:**
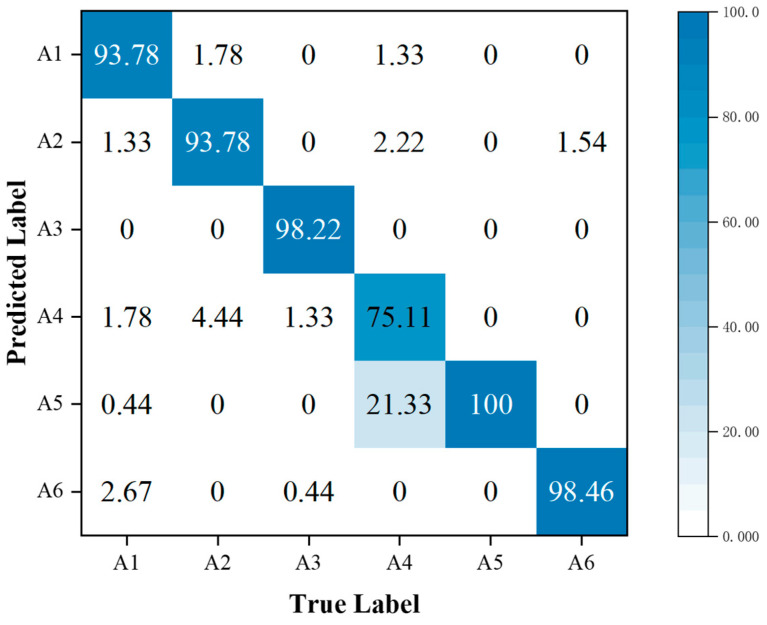
Confusion matrix for classification accuracy of the MFFBN model.

In Figure 10, it is shown that fusing multi-domain features has higher accuracy in HAR for standing up, drinking, and falling compared to the other three activities. Compared to SFN, the accuracy is significantly higher for standing up and drinking activities. However, the recognition accuracy for picking up an object is lower, and the misclassification rate as drinking is higher. It is observed that there are similarities in the 2-D domain spectra of different activities. For instance, activities such as sitting down, standing up, picking up an object, and drinking are all stationary movements and exhibit similar RT maps. Among them, picking up an object and drinking have the highest misclassification rate of 23.11%. Similarly, picking up an object and drinking, which are both bending activities, have similar DT maps. Among them, picking up an object and drinking have the highest misclassification rate of 12.44%. As both the RT and DT maps of these two activities are prone to misclassification, it is evident that the fusion of these two features results in the highest misclassification rate of 21.33% for these two activities. This indicates that directly combining the two 2-D domain spectra features using the proposed multi-domain feature baseline network does not reduce the misclassification rate for these two activities and therefore does not improve the recognition accuracy of the picking up an object activity. However, the subsequent experiments involve fusing the MAFM and the MFL functions to address this issue.

#### 5.2.3. Ablation Experiment

To verify the effectiveness of the MAFM and the MFL function, we conducted the following ablation experiments: (1) introducing the MAFM into the MFFBN model, which is equivalent to the MFAFN model; (2) introducing the MFL function into the MFFBN model; (3) introducing the MFL function into the MFAFN model.

Table 3 shows that in the MFFBN model, with the use of TR and TD maps for feature fusion, the accuracy rate reached 93.1%. Incorporating the MAFM increased the accuracy rate by 3.34%, and adding the MFL function increased it by 2.2%. Adding the MAFM to the MFFBN model achieves higher accuracy growth than the MFL function. Moreover, when both the MAFM and the MFL function were included, the accuracy rate was more significant, with an increase of 4.48%.

**Figure 11:**
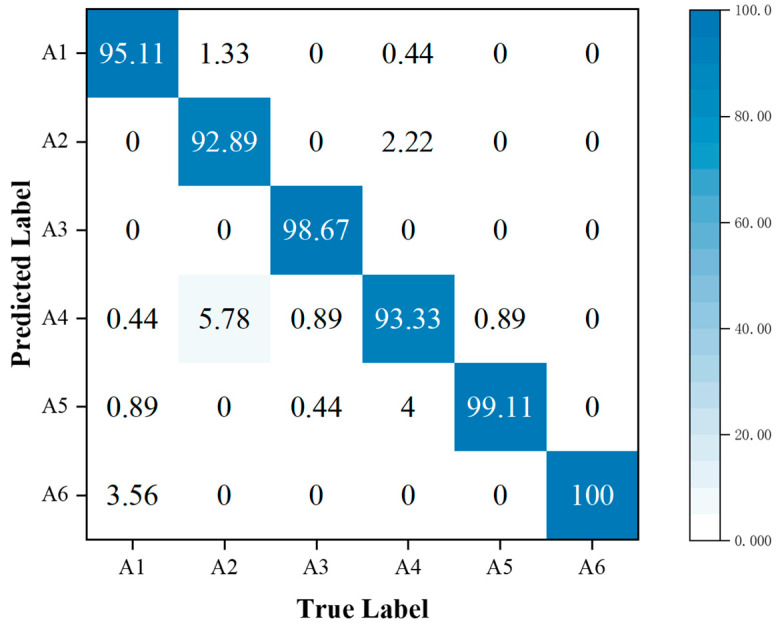
Confusion matrix for classification accuracy of the MFFBN model with the MAFM.

By comparing Figure 11 with Figure 10, we observe that the incorporation of the MAFM into the MFFBN model improved the model’s recognition accuracy for walking, standing up, picking up an object, and falling. The most significant improvement was in the recognition accuracy of picking up an object activity, which increased by 18.22%. This improvement was mainly due to a decrease in the misjudgment rate of picking up an object activity as drinking, which decreased by 17.33%.

**Figure 12:**
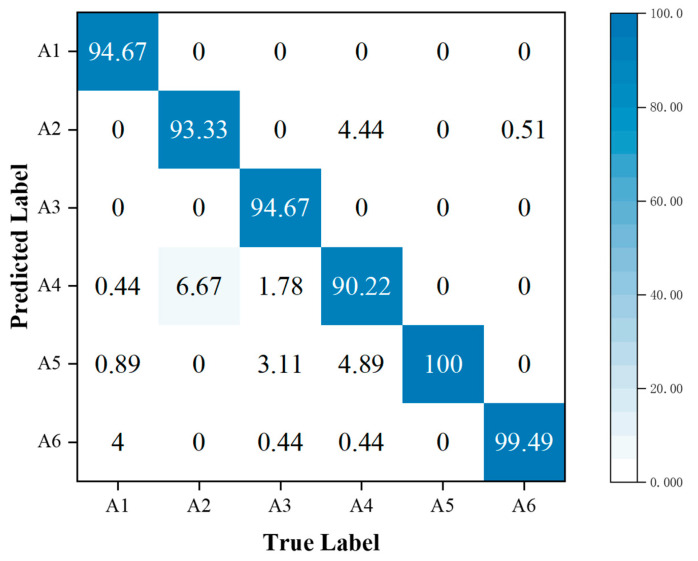
Confusion matrix for classification accuracy of the MFFBN model with the MFL function.

A comparison of Figure 12 with Figure 10 revealed that using the MFL function in the MFFBN model improved recognition accuracy for walking, picking up an object, and falling activities. Notably, the accuracy of picking up an object activity showed a significant increase of 15.11%.

**Figure 13:**
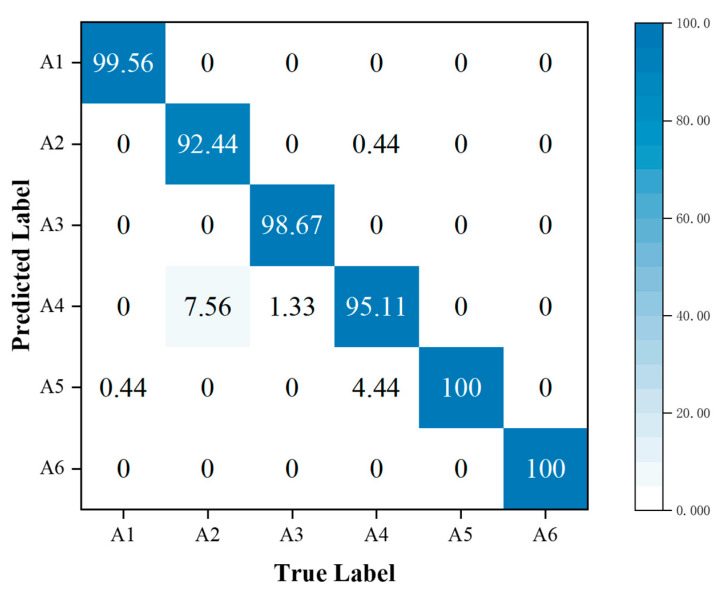
Confusion matrix for classification accuracy of the MFAFN model with the MFL function.

When comparing Figure 13 with Figure 10, we observe that using the MFL function in the MFAFN model, the model’s recognition accuracy for sitting-down activity increased by 1.34%. The recognition accuracy of the drinking activity remained 100% unchanged, and the remaining four activities were improved. The highest recognition accuracy improvement was achieved for the picking up an object activity, which reached 20%.

By applying the framework of our proposed multi-domain fusion hybrid neural network model, we found that combining the two domains leverages their respective strengths. The results show that the information from each domain can complement each other, and fusion can obtain comprehensive information about human activity from radar signals, reducing classification error rates and effectively improving the accuracy of HAR. Besides, the MFL function can also effectively improve the activity recognition accuracy of multi-domain feature fusion networks.

**Figure 14:**
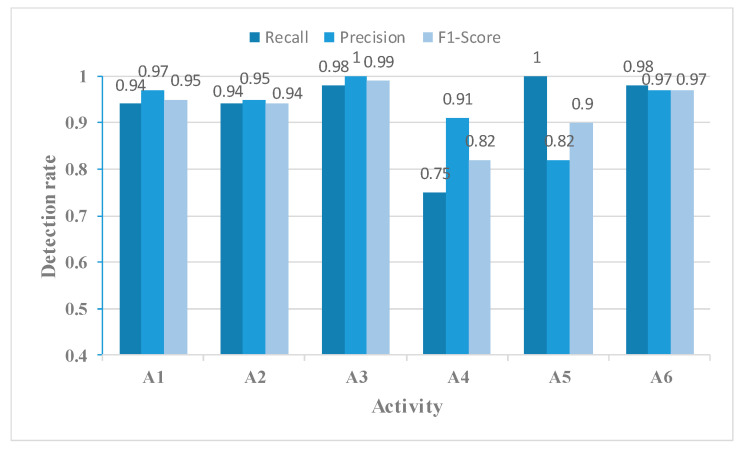
The Recall, Precision, and F1-Score for each activity using the MFFBN model.

**Figure 15:**
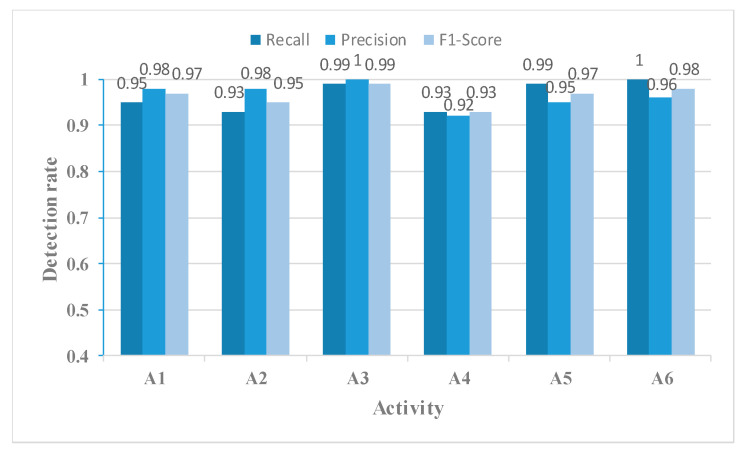
The Recall, Precision, and F1-Score for each activity using the MFAFN model.

**Figure 16:**
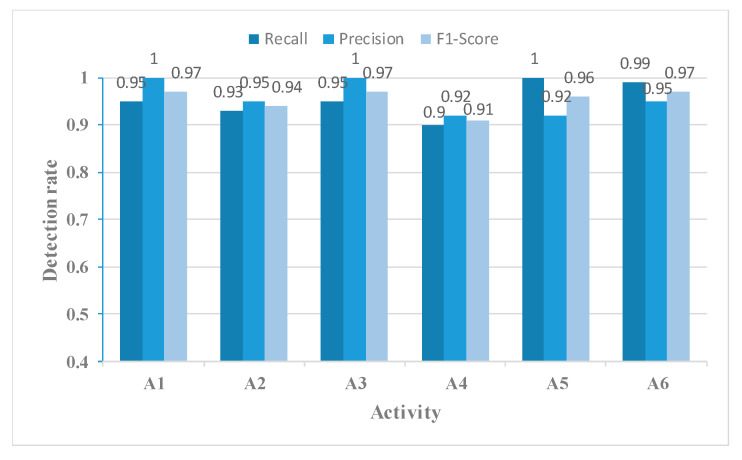
The Recall, Precision, and F1-Score for each activity using the MFFBN model with the MFL function.

**Figure 17:**
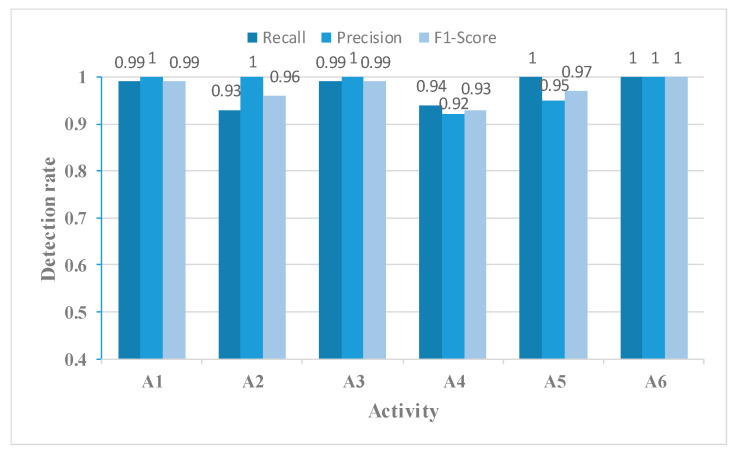
The Recall, Precision, and F1-Score for each activity using the MFAFN model with the MFL function.

Figure 14, Figure 15, Figure 16 and Figure 17 show the Recall, Precision, and F1-Score for each activity classified using the four networks in Table 3. The MFAFN model with the MFL function has the highest Recall for five activity groups: walking, standing up, picking up an object, drinking, and falling. Although the MFFBN has the highest Recall for sitting down, the MFAFN model with the MFL function has only 0.01 lower Recall than the highest value. 

Precision represents the proportion of true positive samples among the samples classified as positive by the classifier. A lower precision indicates a higher probability of misclassifying other samples as positive for that particular sample. It can be observed that the MFFBN model has the lowest Precision for the drinking activity, which is only 0.82, indicating a high probability of misclassifying other activities as drinking. The MFAFN model with the MFL function has a 0.13 increase in Precision for the drinking activity.

Regarding F1-Score, only the MFAFN model with the MFL function achieves a Recall of 1 for the falling activity, indicating accurate recognition without misclassifying other activities as falling. The F1-Score for picking up an object and drinking activities is the lowest in the MFFBN model, with only 0.82 and 0.90, respectively, while the MFAFN model with the MFL function improves by 0.11 and 0.07, respectively.

**Figure 18:**
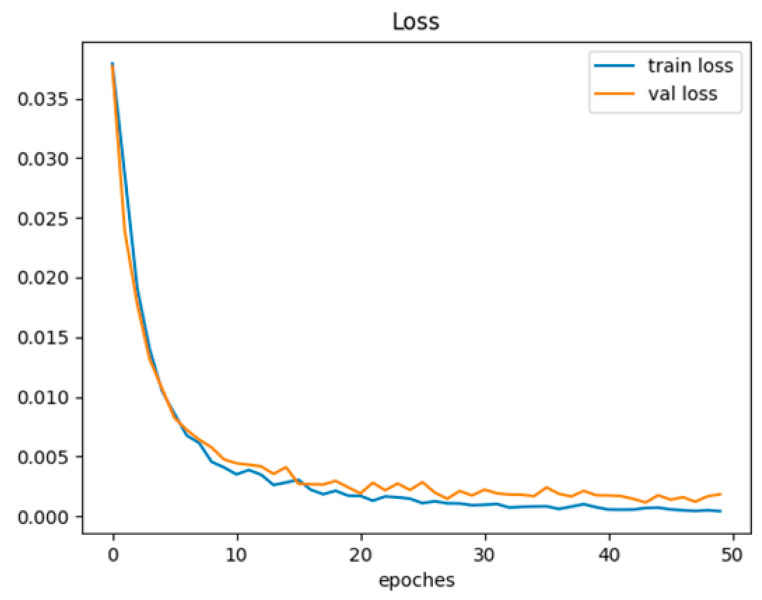
The MFAFN model with the MFL function training and validation loss function.

Figure 18 shows the changes in training and validation losses of the MFAFN model with the MFL function concerning iteration times. This is the loss curve obtained during the initial debugging of the model using a training set, validation set, and test set ratio of 6:2:2, the actual numbers of samples for training, validation, and testing are as follows: 810 samples, 264 samples, and 264 samples. The final experimental results are based on the results of five-fold cross-validation. The results indicate that after 30 epochs, the training and validation loss functions tend to stabilize, indicating convergence of the model. Specifically, the training loss remains stable at 0.001, indicating that the model has effectively learned the training data without overfitting. Overall, the convergence and stability of this model demonstrate the effectiveness of using the MFAFN model with the MFL function to recognize human activities accurately.

#### 5.2.4. Noise Sensitivity Analysis

**Figure 19:**
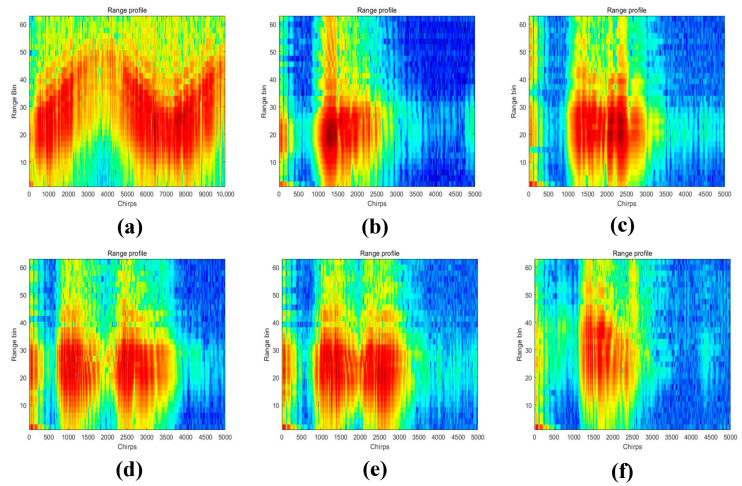
TR maps for six human activities with the threshold set to 80 dB. (**a**) Walking. (**b**) Sitting down. (**c**) Standing up. (**d**) Picking up an object. (**e**) Drinking. (**f**) Falling.

When plotting the 2-D domain spectrograms, we set the values below a threshold to zero in the data matrix. Specifically, when plotting the RT maps, we set amplitudes below 60 dB to zero. To demonstrate the impact of different noise levels on the output results, we compared the experimental results with a threshold set at 80 dB. Figure 19 shows the RT maps for walking, sitting down, standing up, picking up an object, drinking, and falling, with a threshold of 80 dB.

**Figure 20:**
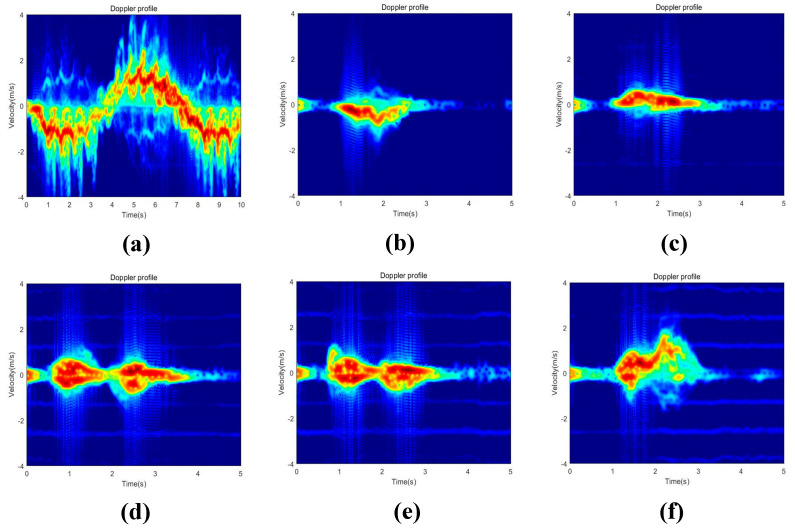
TD maps for six human activities with the threshold set to 60 dB. (**a**) Walking. (**b**) Sitting down. (**c**) Standing up. (**d**) Picking up an object. (**e**) Drinking. (**f**) Falling.

Similarly, when plotting the DT maps, we set amplitudes below 40 dB from the maximum value to zero. To demonstrate the impact of different noise levels on the output results, we compared the experimental results with a threshold set at 60 dB. Figure 20 shows the DT maps for walking, sitting down, standing up, picking up an object, drinking, and falling, with a threshold of 60 dB. 

**Figure 21:**
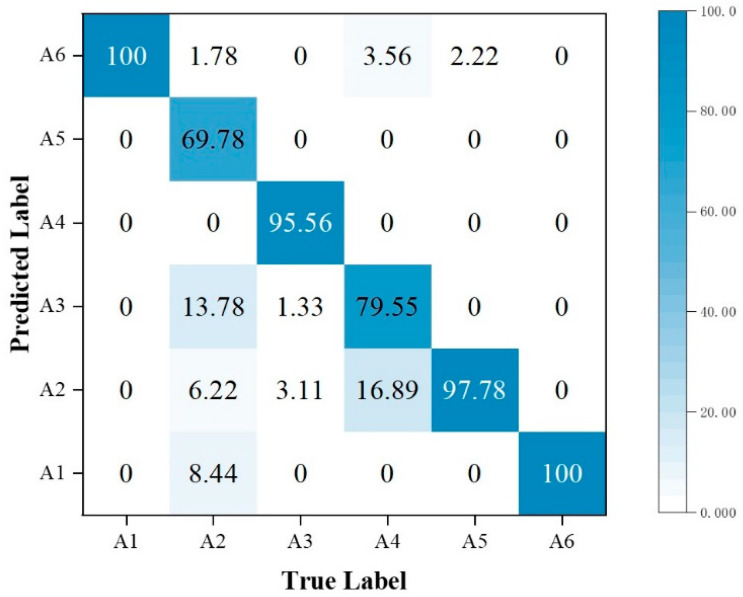
Confusion matrix for classification accuracy of the MFAFN model with the MFL function under different noise environments.

In addition, we used these data as a test set, consisting of 264 RT and DT maps each. We evaluated the performance of the trained model using the MFAFN model with the MFL function and achieved an accuracy of 90.23%. The confusion matrix results are shown in Figure 21.

Comparing Figure 21 with Figure 13, it can be seen that the recognition accuracy for the sitting activity decreased by 22.66%, while the picking up an object activity decreased by 15.56%. This indicates that noise has some effect on both activities. In addition, the recognition accuracy for the sitting down, drinking, and falling activities changed less, indicating that noise had the least effect on these three activities.

#### 5.2.5. Comparison with Other HAR Methods

This section compares the proposed algorithm with the latest HAR methods applied to the same dataset, including [11,17,27,28,31,38]. According to Table 4, the proposed approach outperforms the recent studies with an accuracy improvement ranging from 0.93% to 5.58%.

Among them, refs. [11,17] are single-domain networks that utilize TD maps as inputs for HAR. In [11], three deep learning methods were employed: LSTM, Bi-LSTM, and CNN. The CNN consisted of four convolutional layers, four maximum pooling layers, and four dense layers. PCA and data augmentation methods were combined with CNN to achieve the highest recognition accuracy of 95.3%. In [17], ResNet was utilized to classify human activities, achieving 100% recognition accuracy for walking and falling, while the MFAFN performed better for standing up, picking up an object, and drinking.

In contrast, refs. [27,28,31,38] are multi-domain feature fusion networks. In [38], feature fusion was achieved by incorporating TD maps from different range locations. Despite the differences in selected ranges, the accuracy could be improved by combining the features. The literature [31] obtained an accuracy of 96.65% using a fusion of hand-crafted (H) features and DT graph features extracted with CNN. In [27], a fusion method using the Chi-Square algorithm (CSA) was employed to achieve 92% accuracy in recognizing human activities by fusing features from multiple domains, including spectrogram (µD), CVD, and TR. However, the method was only 76.9% and 84.6% accurate in classifying the activities of picking up an object and drinking, respectively. Despite the potential of multi-domain feature fusion in enhancing activity recognition accuracy, no effective measures were taken to address the issue of confusable activities. In [28], 1-D CNN and LSTM were used to extract features for TR and TD maps, while 2-D CNN pulled features for RD maps, considering different 2-D spectral characteristics. However, its accuracy in fusing TR and TD maps was only 92.24%. The accuracy of combining the three features reached 93.39%, and both this network and the MFAFN model with the MFL function achieved 100% identification accuracy in falling activity. Nevertheless, our method outperformed this multi-domain feature fusion network in all three actions: sitting down, picking up an object, and drinking. 

We utilized TR and TD maps and employed the MFAFN model to fuse these features effectively. Additionally, we used the MFL function to enhance confusable sample weights, resulting in the MFAFN model that achieved a classification accuracy of 97.58%. Notably, our method outperforms the latest methods mentioned in the literature, particularly concerning two activities: picking up an object and drinking. Compared to the literature [27], our process improves the accuracy of these activities by 17.32% and 15.4%, respectively. Compared to the literature [28], our approach improves the accuracy of these activities by 7.73% and 18.33%, respectively. Our method can effectively improve the classification accuracy of confusable samples.

## 6. Conclusions

This paper presents a multi-domain feature fusion network based on an FMCW radar sensor that improves the accuracy of HAR by fusing the TR and TD domains of human activities. Our proposed MFAFN model and the MFL function are evaluated on the University of Glasgow dataset, and the extensive experiments demonstrate their effectiveness, achieving an accuracy of 97.58%. Our approach improves the accuracy of HAR by 5.46% compared to single-domain feature networks using TD maps, and effectively improves classification results for confusable samples by up to 18.22% compared to the MFFBN. In reference [28], a multi-domain feature fusion network was also employed to combine TR and TD maps. Experimental results demonstrated that fusing these two types of 2-D domain spectra effectively improved the accuracy of HAR. However, this method exhibited a higher misclassification rate for confusing activities such as picking up an object or drinking. To address this issue, we introduce MAFM and MFL functions in the MFFBN, which effectively enhance the accuracy of recognizing confusing activities. In conclusion, our method can achieve better results in confusable activity recognition.

## Figures and Tables

**Table 1:** Comparison of different focus loss factors.

Parameters	γ = 1.5	γ = 2	γ = 2.5
Add the MAFM and the MFL function	97.16%	97.58%	97.08%

**Table 2:** Comparison of accuracy between the MFFBN and the SFN models.

Method	Data Type	Accuracy (%)
The SFN model	TR	75.08
The SFN model	TD	92.12
The MFFBN model	TD, TR	93.1

**Table 3:** Ablation experiment.

Method	Data Type	Accuracy (%)
The MFFBN model	TD, TR	93.1
Add the MAFM	TD, TR	96.44
Add the MFL function	TD, TR	95.3
Add the MAFM and the MFL function	TD, TR	97.58

**Table 4:** MFAFN model and other HAR methods for the same dataset.

Method	Type of Data	Model Type	Accuracy (%)
[27]	µD, CVD, TR	CSA	92
[38]	RTD	RD-CNN	92.33
[28]	TR, TD, RD	CNN and LSTM	93.39
[11]	TD	CNN and PCA	95.3
[17]	TD	ResNet	96
[31]	H, TD, PD	CNN	96.65
Ours	TR, TD	CNN and SMAN	97.58

## Data Availability

The original datasets are publicly available from http://researchdata.gla.ac.uk/848/ (accessed on 22 May 2023).

## Appendix A

The abbreviations commonly used in this paper are shown in Table A1.

**Table A1:** Table of abbreviations.

Full Name	Abbreviation
Human Activity Recognition	HAR
Multi-domain Feature Fusion Network	MFFN
Multi-domain Feature Fusion Baseline Network	MFFBN
Multi-feature Attention Fusion Module	MAFM
Multi-classification Focus Loss	MFL
Multi-domain Feature Attention Fusion Network	MFAFN
Time-Doppler	TD
Time-Range	TR

## References

[1] Gorji A., Khalid H.-U., Bourdoux A., Sahli H. (2021). On the Generalization and Reliability of Single Radar-Based Human Activity Recognition. IEEE Access.

[2] He Y., Li X., Jing X. (2019). A Mutiscale Residual Attention Network for Multitask Learning of Human Activity Using Radar Micro-Doppler Signatures. Remote Sens..

[3] Shahmohammadi F., Hosseini A., King C.E., Sarrafzadeh M. Smartwatch based activity recognition using active learning. Proceedings of the 2017 IEEE/ACM International Conference on Connected Health: Applications, Systems and Engineering Technologies (CHASE), IEEE.

[4] Habib S., Hussain A., Albattah W., Islam M., Khan S., Khan R.U., Khan K. (2021). Abnormal Activity Recognition from Surveillance Videos Using Convolutional Neural Network. Sensors.

[5] Li X., He Y., Jing X. (2019). A Survey of Deep Learning-Based Human Activity Recognition in Radar. Remote Sens..

[6] Coelho Y.L., dos Santos F.D.A.S., Frizera-Neto A., Bastos-Filho T.F. (2021). A Lightweight Framework for Human Activity Recognition on Wearable Devices. IEEE Sens. J..

[7] Alrashdi I., Siddiqi M.H., Alhwaiti Y., Alruwaili M., Azad M. (2021). Maximum Entropy Markov Model for Human Activity Recognition Using Depth Camera. IEEE Access.

[8] Li G., Zhang R., Ritchie M., Griffiths H. (2018). Sparsity-driven micro-Doppler feature extraction for dynamic hand gesture recog-nition. IEEE Trans. Aerosp. Electron. Syst..

[9] Chen Z., Li G., Fioranelli F., Griffiths H. (2018). Personnel Recognition and Gait Classification Based on Multistatic Micro-Doppler Signatures Using Deep Convolutional Neural Networks. IEEE Geosci. Remote Sens. Lett..

[10] Chakraborty M., Kumawat H.C., Dhavale S.V. (2022). DIAT-RadHARNet: A Lightweight DCNN for Radar Based Classification of Human Suspicious Activities. IEEE Trans. Instrum. Meas..

[11] Taylor W., Dashtipour K., Shah S.A., Hussain A., Abbasi Q.H., Imran M.A. (2021). Radar Sensing for Activity Classification in Elderly People Exploiting Micro-Doppler Signatures Using Machine Learning. Sensors.

[12] Senigagliesi L., Ciattaglia G., Disha D., Gambi E. Classification of Human Activities based on Automotive Radar Spectral Images Using Machine Learning Techniques: A Case Study. Proceedings of the 2022 IEEE Radar Conference (RadarConf22).

[13] Li Y., Li Z., Wang Y., Xie G., Lin Y., Shen W., Jiang W. (2023). Improving the Performance of RODNet for MMW Radar Target Detection in Dense Pedestrian Scene. Mathematics.

[14] Abdu F.J., Zhang Y., Deng Z. (2022). Activity Classification Based on Feature Fusion of FMCW Radar Human Motion Micro-Doppler Signatures. IEEE Sens. J..

[15] Sun M., Xu Z., Sun B., Zhang S. FMCW Multi-Person Action Recognition System Based on Point Cloud Nearest Neighbor Sam-pling Algorithm. Proceedings of the 2021 4th International Conference on Pattern Recognition and Artificial Intelligence (PRAI).

[16] Huang X., Ding J., Liang D., Wen L. (2020). Multi-Person Recognition Using Separated Micro-Doppler Signatures. IEEE Sens. J..

[17] Saeed U., Shah S.Y., Shah S.A., Ahmad J., Alotaibi A.A., Althobaiti T., Ramzan N., Alomainy A., Abbasi Q.H. (2021). Discrete human activity recognition and fall detection by combining FMCW RADAR data of heterogeneous environments for independent assistive living. Electronics.

[18] Zhu J., Chen H., Ye W. (2020). A Hybrid CNN–LSTM Network for the Classification of Human Activities Based on Micro-Doppler Radar. IEEE Access.

[19] Shrestha A., Li H., Le Kernec J., Fioranelli F. (2020). Continuous Human Activity Classification from FMCW Radar with Bi-LSTM Networks. IEEE Sens. J..

[20] Li H., Mehul A., Le Kernec J., Gurbuz S.Z., Fioranelli F. (2020). Sequential Human Gait Classification with Distributed Radar Sensor Fusion. IEEE Sens. J..

[21] Gorji A., Gielen T., Bauduin M., Sahli H., Bourdoux A. A Multi-radar Architecture for Human Activity Recognition in Indoor Kitchen Envi-ronments. Proceedings of the 2021 IEEE Radar Conference (RadarConf21).

[22] Li H., Shrestha A., Heidari H., Le Kernec J., Fioranelli F. (2018). A Multisensory Approach for Remote Health Monitoring of Older People. IEEE J. Electromagn. RF Microw. Med. Biol..

[23] Li H., Shrestha A., Heidari H., Le Kernec J., Fioranelli F. (2018). Magnetic and Radar Sensing for Multimodal Remote Health Monitoring. IEEE Sens. J..

[24] Li H., Shrestha A., Heidari H., Le Kernec J., Fioranelli F. (2019). Bi-LSTM network for multimodal continuous human activity recognition and fall detec-tion. IEEE Sens. J..

[25] Arab H., Ghaffari I., Chioukh L., Tatu S.O., Dufour S. (2022). A Convolutional Neural Network for Human Motion Recognition and Classification Using a Millimeter-Wave Doppler Radar. IEEE Sens. J..

[26] Zhang X., Abbasi Q.H., Fioranelli F., Romain O., Le Kernec J. (2022). Elderly Care-Human activity recognition using radar with an open dataset and hybrid maps. Body Area Networks. Smart IoT and Big Data for Intelligent Health Management, Proceedings of the 16th EAI International Conference, BODYNETS 2021, Virtual Event, 25–26 October 2021.

[27] Li Z., Fioranelli F., Yang S., Zhang L., Romain O., He Q., Cui G., Le Kernec J. Multi-domains based human activity classification in radar. Proceedings of the IET International Radar Conference (IET IRC 2020).

[28] Ding W., Guo X., Wang G. (2021). Radar-Based Human Activity Recognition Using Hybrid Neural Network Model with Multidomain Fusion. IEEE Trans. Aerosp. Electron. Syst..

[29] Wang X., Guo S., Chen J., Gui G. (2022). GCN-Enhanced Multi-domain Fusion Network for Through-wall Human Activity Recognition. IEEE Geosci. Remote Sens. Lett..

[30] Bai X., Hui Y., Wang L., Zhou F. (2019). Radar-Based Human Gait Recognition Using Dual-Channel Deep Convolutional Neural Network. IEEE Trans. Geosci. Remote Sens..

[31] Jia M., Li S., Le Kernec J., Yang S., Fioranelli F., Romain O. Human activity classification with radar signal processing and machine learning. Proceedings of the 2020 International conference on UK-China Emerging Technologies (UCET).

[32] Zhao Y., Hu W. CentralNet Method for Human motion Recognition Based on Multi-feature Fusion of Millimeter Wave Radar. Proceedings of the 2021 IEEE International Conference on Signal Processing, Communications and Computing (ICSPCC).

[33] Chen P., Jian Q., Wu P., Guo S., Cui G., Jiang C., Kong L. (2021). A Multi-Domain Fusion Human Motion Recognition Method Based on Lightweight Network. IEEE Geosci. Remote Sens. Lett..

[34] Gao Y., Zhou Y., Wang Y., Zhuo Z. (2020). Narrowband Radar Automatic Target Recognition Based on a Hierarchical Fusing Network with Multidomain Features. IEEE Geosci. Remote Sens. Lett..

[35] Helen Victoria A., Maragatham G. (2021). Activity recognition of FMCW radar human signatures using tower convolutional neural networks. Wirel. Netw..

[36] Chen Y., Wang W., Liu Q., Sun Y., Tang Z., Zhu Z. Human activity classification with radar based on Multi-CNN information fusion. Proceedings of the IET International Radar Conference (IET IRC 2020).

[37] Jokanovic B., Amin M., Erol B. Multiple Joint-Variable Domains Recognition of Human Motion. Proceedings of the 2017 IEEE Radar Conference.

[38] Kim W.Y., Seo D.H. (2022). Radar-Based Human Activity Recognition Combining Range–Time–Doppler Maps and Range-Distributed-Convolutional Neural Networks. Proceedings of the IEEE Transactions on Geoscience and Remote Sensing.

[39] Du C., Zhang L., Sun X., Wang J., Sheng J. (2020). Enhanced Multi-Channel Feature Synthesis for Hand Gesture Recognition Based on CNN with a Channel and Spatial Attention Mechanism. IEEE Access.

[40] Campbell C., Ahmad F. Attention-augmented convolutional autoencoder for radar-based human activity recognition. Proceedings of the 2020 IEEE International Radar Conference (RADAR).

[41] Fairchild D.P., Narayanan R.M. (2014). Classification of human motions using empirical mode decomposition of human micro-Doppler signatures. IET Radar Sonar Navig..

[42] Krizhevsky A., Sutskever I., Hinton G.E. (2017). Imagenet classification with deep convolutional neural networks. Commun. ACM.

[43] Simonyan K., Zisserman A. (2014). Very deep convolutional networks for large-scale image recognition. arXiv.

[44] Szegedy C., Liu W., Jia Y., Sermanet P., Reed S., Anguelov D., Erhan D., Vanhoucke V., Rabinovich A. Going deeper with convolutions. Proceedings of the IEEE Conference on Computer Vision and Pattern Recognition.

[45] Hu J., Shen L., Sun G. Squeeze-and-excitation networks. Proceedings of the IEEE Conference on Computer Vision and Pattern Recognition.

[46] Liu W., Chen L., Chen Y. (2018). Age classification using convolutional neural networks with the multi-class focal loss. Materials Science and Engineering.

[47] Shah S.A., Fioranelli F. Human activity recognition: Preliminary results for dataset portability using FMCW radar. Proceedings of the 2019 International Radar Conference (RADAR).

